# Efficacy of a workplace osteoporosis prevention intervention: a cluster randomized trial

**DOI:** 10.1186/s12889-016-3506-y

**Published:** 2016-08-24

**Authors:** Ai May Tan, Anthony D. LaMontagne, Dallas R. English, Peter Howard

**Affiliations:** 1 McCaughey Centre: VicHealth Centre for the Promotion of Mental Health & Community Wellbeing, Melbourne School of Population and Global Health, University of Melbourne, Melbourne, VIC 3010 Australia; 2 Centre for Population Health Research, Building BC, Level 3, School of Health & Social Development, Deakin University, Burwood, VIC 3125 Australia; 3 Centre for Epidemiology and Biostatistics, Melbourne School of Population and Global Health, University of Melbourne, Melbourne, VIC 3010 Australia; 4 Cancer Epidemiology Centre, Cancer Council Victoria, Melbourne, 3004 Australia; 5 Melbourne School of Population and Global Health, University of Melbourne, Melbourne, VIC 3010 Australia

**Keywords:** Osteoporosis prevention, Cluster randomized trial, Premenopausal women, Workplace, Calcium intake, Physical activity

## Abstract

**Background:**

Osteoporosis is a debilitating disease. Adequate calcium consumption and physical activity are the two major modifiable risk factors. This paper describes the major outcomes and efficacy of a workplace-based targeted behaviour change intervention to improve the dietary and physical activity behaviours of working women in sedentary occupations in Singapore.

**Methods:**

A cluster-randomized design was used, comparing the efficacy of a tailored intervention to standard care. Workplaces were the units of randomization and intervention. Sixteen workplaces were recruited from a pool of 97, and randomly assigned to intervention and control arms (eight workplaces in each). Women meeting specified inclusion criteria were then recruited to participate. Workplaces in the intervention arm received three participatory workshops and organization-wide educational activities. Workplaces in the control/standard care arm received print resources. Outcome measures were calcium intake (milligrams/day) and physical activity level (duration: minutes/week), measured at baseline, 4 weeks and 6 months post intervention. Adjusted cluster-level analyses were conducted comparing changes in intervention versus control groups, following intention-to-treat principles and CONSORT guidelines.

**Results:**

Workplaces in the intervention group reported a significantly greater increase in calcium intake and duration of load-bearing moderate to vigorous physical activity (MVPA) compared with the standard care control group. Four weeks after intervention, the difference in adjusted mean calcium intake was 343.2 mg/day (95 % CI = 337.4 to 349.0, *p* < .0005) and the difference in adjusted mean load-bearing MVPA was 55.6 min/week (95 % CI = 54.5 to 56.6, *p* < .0005). Six months post intervention, the mean differences attenuated slightly to 290.5 mg/day (95 % CI = 285.3 to 295.7, *p* < .0005) and 50.9 min/week (95 % CI =49.3 to 52.6, *p* < .0005) respectively.

**Conclusion:**

This workplace-based intervention substantially improved calcium intake and load-bearing moderate to vigorous physical activity 6 months after the intervention began.

**Trial registration:**

Australia New Zealand Clinical Trial Registry ACTRN12616000079448. Registered 25 January 2016 (retrospectively registered)

**Electronic supplementary material:**

The online version of this article (doi:10.1186/s12889-016-3506-y) contains supplementary material, which is available to authorized users.

## Background

Calcium intake and physical activity are modifiable risk factors for osteoporosis operating through the maintenance of bone mass and skeletal integrity [1, 2]. Evidence suggests that physical activity and calcium intake can affect not just bone mineral density, but also risk of osteoporotic fractures [3, 4]. Prospective longitudinal studies have estimated that 23 % of osteoporosis is attributable to physical inactivity [3] and that almost 10 % of osteoporotic fractures are attributable to low dietary calcium intake [4]. This suggests that there are substantial preventable fractions in the order of 10–20 % for osteoporosis and osteoporotic fractures, and that efforts to develop intervention strategies to improve calcium intake and physical activity to achieve this are warranted.

Previous research suggests that health education or health promotion interventions have the potential to influence selected health behaviours that affect bone health [5–8]. Interventions designed to enhance knowledge and self-efficacy showed increased calcium intake in the short-term [5, 7, 9–13]. Physical activity outcomes, however, were less positive [5, 11, 14, 15]. The majority of population-based interventions addressing osteoporosis prevention have not referenced past evidence to determine the level of behaviour change required to make an impact on the disease and its consequences [5–7, 9–17]. Some interventions consisted of one-off information sessions or print resource distribution [8, 10, 11, 17] and the majority did not appear to have designed unique intervention strategies to address dietary behaviours and physical activity separately [6, 10–17].

The workplace has not been explored as a platform for osteoporosis prevention interventions. Workplaces with predominantly sedentary employees present unique opportunities for osteoporosis prevention, as occupational sitting has been associated with low bone mineral density [18]. Women in sedentary occupations are a priority group for osteoporosis prevention, as being both female and sedentary are independent risk factors for low bone mass and osteoporosis.

Existing evidence points to unrealized potential in both intervention design and use of the workplace setting in osteoporosis prevention. This study improves on previous research and practice as follows:

1. To our knowledge, it is the first to address dietary and physical activity components each with unique intervention strategies in the context of osteoporosis prevention.
2. The intervention strategy for both behaviours was based on self-efficacy theory, focusing on behavioural rather than cognitive strategies.
3. The utilization of a workplace platform for osteoporosis prevention.
4. The specification of intervention outcomes and levels required to constitute meaningful change in terms of the prevention of osteoporosis and osteoporotic fractures.
5. The use of a strong study design to estimate the benefit of the intervention over and above standard care (current practice).

This cluster randomized trial tests the hypothesis that a tailored and self-efficacy focused workplace intervention is more efficacious than standard care (print resources) in increasing calcium intake and physical activity levels.

## Methods

The full study protocol has been previously reported [19]. This was a two-arm, cluster randomized trial. Clusters were workplaces that were randomly assigned to receive either i) tailored workplace-based intervention or ii) print resources (standard care control arm).

### Sample size calculation

Sample size calculations took into consideration the cluster randomized design, incorporating design effects based on a minimum cluster size of 20 individuals and published or estimated intraclass correlation coefficients (ICC) for each of the outcomes, as detailed in a previously published protocol for the trial. [19] There were 16 worksites/clusters randomized to intervention (8 sites) and control (8 sites) by a statistician who was blinded to the identities of the worksites [19].

### Sampling frame, recruitment and study sample

Workplaces were sampled from a database of workplaces that were recipients of a 2003 Singapore Health Award. These workplaces would have demonstrated commitment to promoting employee health to receive this national award, hence the characterization of this trial as assessing efficacy rather than effectiveness.

### Workplace (cluster) inclusion criteria

- Workplaces in sectors or industries that were primarily office based and sedentary in nature, such as government administration departments and finance;
- Workplaces that were able to recruit at least 30 female employees engaged in desk-based jobs (sitting for at least 50 % of working hours); and
- Agreement to permit up to 10 h of paid work time during the course of the study (12 months) for the recruited employees to participate in pre-post data collection and intervention activities.

### Eligibility for recruitment of employees within selected workplaces:

- Being female;
- Age 25–49 years of age; and
- Being in a sedentary job (at least 50 % of work hours seated)

### Exclusion criteria were:

- Being pregnant or lactating;
- Diagnosed osteoporosis;
- Diagnosed kidney problems; and
- Participation in another health program that addressed diet and/or physical activity.

### Outcome measures

Calcium intake was measured using a 3-day diet record which involved each participant keeping a detailed written record of the foods and beverages consumed over two representative weekdays and one representative weekend day. Three day recording was selected as recording periods of more than 3 or 4 days were reported to be unreliable due to respondent fatigue [20]. Details of dietary record procedures have been previously published [19].

Physical activity was measured using the EPIC Norfolk Physical Activity Questionnaire 2 (EPAQ-2): a self-reported questionnaire designed to measure different sub-dimensions of physical activity including load-bearing activity of relevance to osteoporosis prevention [21]. The EPAQ-2 has been validated for use in large-scale epidemiological studies [21].

The content of the EPAQ-2 was assessed for cultural appropriateness and ease of reading [19]. Minor modifications were made to the list of recreation activities. Activities that were not relevant to the local context, such as “digging, shoveling or chopping wood” were removed, and replaced with common local activities not included in the version developed for use in Europe, such as practicing Tai Chi.

### Self-efficacy data and other information

As previously described [19], data on calcium intake and exercise self-efficacy scores were collected using the osteoporosis self-efficacy scale developed and evaluated by Horan et al. in 1998 [22]. The content of the questionnaire was assessed for appropriateness to local context by a panel that included experts internal and external to the Health Promotion Board (Singapore). It was also validated for internal consistency and test-retest repeatability as previously described [19]. Socio-demographic and additional health information was collected at baseline using a questionnaire [19].

### Intervention description

Subjects from workplaces assigned to the intervention group received three intensive workshops with strong focus on behavioural strategies guided by Bandura’s Self–Efficacy Model. [19, 23] The workshop design focused on individual goal setting and on participatory skill-building activities, goal-setting exercises, peer support and problem-solving discussions to attain individual goals and overcome individual barriers [19]. The intervention addressed diet and physical activity as different entities that required different behavioural strategies. Though guided by the same principles, the workshops for diet and physically activity were unique in the nature and design of their activities [19].

### Calcium intake intervention

Past studies that reported positive outcomes for calcium intake describe participatory activities that target the participants’ lifestyles and tastes and incorporation of local food sources [14, 24–26]. Evidence also suggested that the provision of calcium intake feedback might be an effective tool to improve behaviour [27]. These elements were used to guide the development of intervention content. Food sampling, nutrition label reading and group activities with exchange of ideas were key activities in the intervention [19]. The baseline dietary records were inspected to identify common calcium food sources and consumption patterns amongst the study population. This information was utilized to tailor strategies for the intervention [19].

### Physical activity intervention

Intervention targeted uptake of load-bearing moderate to vigorous level of physical activity (MVPA) and resistance training exercises, which can affect bone mineral density, risk of osteoporosis and risk of fractures [28–31].

Meta-analyses of physical activity interventions emphasize the importance of behavioural interventions, which include goal setting, self-monitoring, physical activity behaviour feedback, exercise prescription and cues [32, 33]. Our study included these elements in the design of our intervention activities. Emphasis was placed on providing opportunities to sample a variety of the targeted physical activities. Intervention activities are detailed in the published protocol for this study [19].

Almost 50 % of Singapore adults cited lack of time as a barrier to leisure time physical activity [34]. Unique strategies were developed for this study to facilitate the attainment of the physical activity goal with minimal disruption to the participants’ routine such as introducing short bouts of exercise breaks (5–10 min) incorporated into home or work routine, which many participants would regard as achievable when time and space were limiting factors. Resources, such as an exercise CD and a 10-min exercise poster with instructions and illustrations were provided.

### Control arm

Participants in control/standard care workplaces received a resource kit with general print resources on bone health and osteoporosis prevention. They received a report with their average calcium intake based on their dietary records but were not provided with recommendations for change. Both groups received information and recommendations about vitamin D.

### Data analysis

This study used a two-stage adjusted analysis based on cluster summaries [35] to allow for adjustment for imbalance in potential confounders between intervention and control groups. This approach adheres to the recommendation of the 2004 CONSORT Statement for cluster randomized trials to account for the clustering effect [35, 36] and is preferred over multilevel/mixed models for studies with small number of clusters [35, 37]. All analyses followed intention-to-treat principles and CONSORT guidelines [36].

**Fig. 1:**
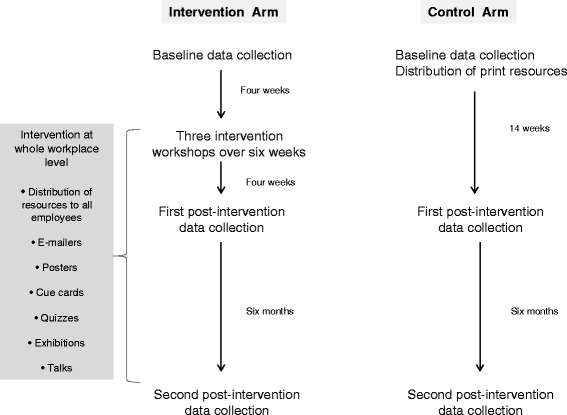
Data collection and intervention timeline

Data from the first and second follow-ups (Fig. 1) were analyzed to compare short-term changes after the intervention and to assess sustainability of any observed changes. The primary dependent variables were calcium intake (milligrams per day) and load-bearing MVPA level (duration in minutes per week). A definition of load-bearing MVPA has been provided in the Additional file 1.

Stage one of the analysis involved linear regression modelling of follow-up outcome measures (as dependent variables) adjusting for baseline outcome measures and potential confounders, ignoring intervention status and cluster. Personal income, education and religion were identified as a priori confounders and were included in regression models individually and in combination. Individuals’ residual outputs, the differences between the observed values and the model predicted values, from each analysis (4 weeks and 6 months follow-ups) were saved to be used in stage two of the analysis.

Stage two entailed analyses of cluster-level summaries to compare differences between the intervention and control groups. Individual residuals calculated at stage one were used to generate cluster means, yielding eight observations for intervention and eight for control sites (or clusters). Independent samples t-tests were used to compare the cluster means for the intervention versus the control groups (*n* = 16).

**Fig. 2:**
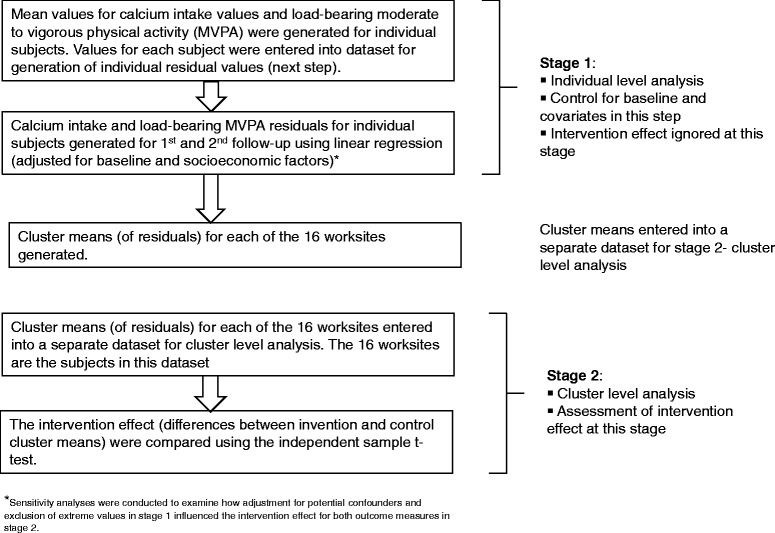
Data analysis flowchart

Figure 2 describes the flow of this two-stage data analysis.

Sensitivity analyses were also conducted to examine how adjustment for potential confounders and exclusion of extreme values influenced the intervention effect for both outcome measures.

Dichotomous analyses were also carried out at the cluster level to examine potential public health implications. This compared the proportion of subjects who achieved daily recommended calcium intake allowance of 800 mg, the proportion who reported nil leisure time load-bearing MVPA and the proportion who achieved at least 60 min of load-bearing MVPA per week, between intervention and control groups.

For comparison with the values employed in the design of the study, post hoc ICCs for calcium intake and load-bearing MVPA were calculated using one-way analysis of variance of all 16 clusters using the equations sourced from Ukoumunne et al. (1999) [38]. Confidence intervals for the ICCs were calculated using the method recommended by Donner (1979) [39].

Most analyses were completed using IBM® SPSS® version 21.0. The calculation of the sample size and the post hoc ICCs were completed using STATA® version 9.0.

## Results

Ninety-seven workplaces were invited to participate in this study. Thirty-seven workplaces responded and the first 16 were recruited and randomly assigned to control or intervention arm, as detailed previously [19]. In the intervention group, four workplaces were government offices, two were tertiary education institutions and two were private companies in property development and publishing. In the control group, four workplaces were government offices, two were quasi-government companies and two were private companies in insurance and internet service.

### Participant characteristics

**Fig. 3:**
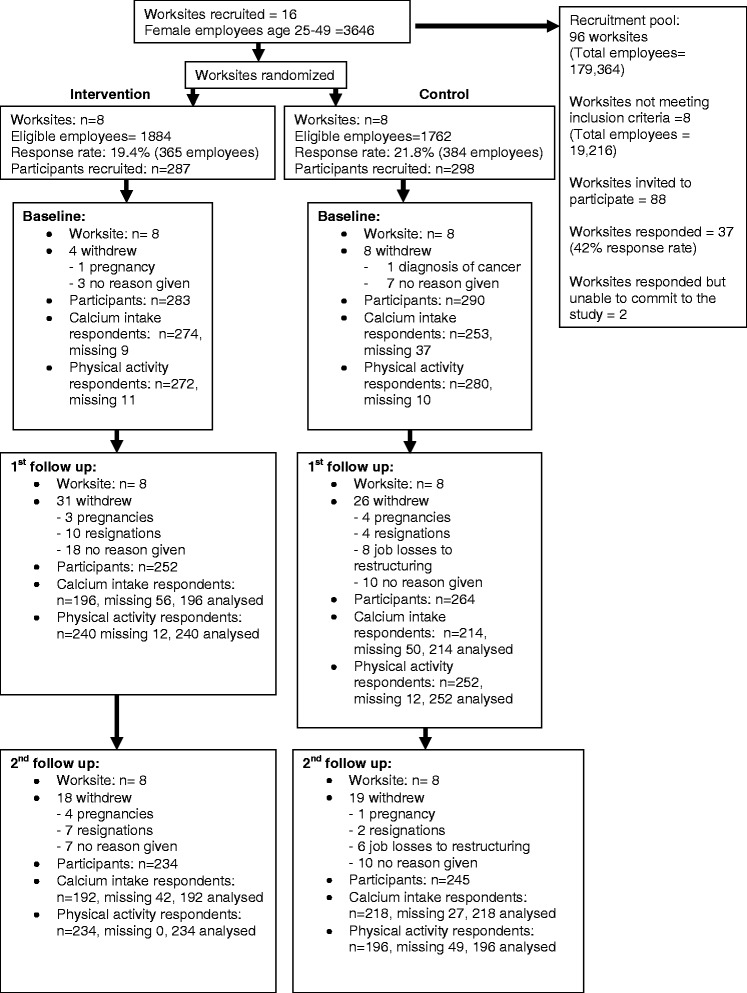
Consort flow diagram for worksites and participants

Five hundred and eighty-five eligible women from 16 workplaces consented to participate, of which 49.1 % (*n* = 287) were in the intervention group and 50.9 % (*n* = 298) in the control group. The baseline characteristics for both groups are displayed in Table 1. Figure 3 displays the consort follow chart.

**Table 1:** Baseline demographic characteristics, calcium intake and load bearing-moderate to vigorous physical activity (MVPA)

Characteristics and measures	Intervention (*n* = 287)	Control (*n* = 298)	*p*-value
Age (mean, SD)	37 (±6.73)	37 (±7.41)	0.972*
Marital status^a^	*n* = 280	*n* = 280	0.859**
Married	181	183	
Single/Divorced/Widowed	99	97	
Personal Income^b^	*n* = 272	*n* = 276	0.001**
<$3000	129	176	
$3000–$4999	97	71	
>$5000	46	29	
Household income^c^	*n* = 247	*n* = 256	0.083**
<$3000	27	38	
$3000–$4999	61	82	
$5000–9999	119	104	
>$10,000	40	82	
Education^d^	*n* = 280	*n* = 279	0.004**
Below tertiary	100	135	
Above tertiary	145	124	
Post-graduate	35	20	
Religion^e^	*n* = 274	*n* = 273	<0.005**
No religion	70	55	
Christianity	98	64	
Buddhism	66	80	
Islam	16	42	
Others	24	32	
Family history of osteoporosis^f^	*n* = 277	*n* = 279	0.825**
Yes	20	21	
No	236	233	
Don’t know	21	25	
Average calcium intake (milligrams per day)	454.8 (±178.04)	462.1 (±202.2)	0.160*
Average duration of load-bearing MVPA^h^ (minutes per week)	59.2 (±94.4)	54.5 (±78.7)	0.194*
Participants meeting RDA^i^ (%)	5.47 (15 out of 274)	6.72 (17 out of 253)	0.550**
Load-bearing MVPA status	*n* = 272	*n* = 280	0.776**
0 minutes	102 (35.5 %)	98 (32.9 %)	
Up to 30 min	42 (14.6 %)	46 (15.4 %)	
30–60 min	30 (10.5 %)	38 (12.8 %)	
60 min or more	98 (34.1 %)	98 (32.9 %)	
Missing	15 (5.2 %)	18 (6 %)	

*P* values calculated from *Independent sample *t*-test; **Chi square using SPSS® (version 21.0)

^a^25 missing cases; ^b^82 missing cases; ^c^38 missing cases; ^d^37 missing cases; ^e^38 missing cases; ^f^29 missing cases; ^g^58 missing cases; ^h^33 missing cases

^i^RDA = Recommended Daily Allowance or Recommended Dietary Allowance

The intervention group reported a higher proportion of participants in the higher personal income and higher education categories. There was also a significant imbalance in stated religious affiliation, which, in the Singapore context, might potentially influence dietary patterns and hence calcium consumption. Thus, personal income, education status and religion were assessed as potential confounders in the analysis.

The baseline calcium intake and baseline load-bearing MVPA duration were similar in both groups (454.8 mg versus 462.1 mg and 59.2 min versus 54.5 min for intervention group and control groups respectively) (Table 1). The proportion of participants who met the RDA was similar in both groups. The proportion of participants in different levels of load-bearing MVPA participation was also similar in both groups.

### Analysis of main outcome measures

#### Calcium intake

At the first follow up (4 weeks), the mean calcium intake was 400.2 mg/day (95 % CI = 395.0 to 405.4) higher for the intervention compared with the control group without cluster-level adjustment for potential confounders. After excluding data more than two standard deviations from the mean, the difference was 355.5 mg/day (95 % CI = 349.4 to 361.7). The exclusion of two standard deviation outliers in a sensitivity analyses (detailed in Additional file 2 “Sensitivity analysis report”) led to the most conservative estimates of the intervention effect, hence only these results are presented in Table 2. After adjustment for potential confounders, the mean difference was 343.2 mg/day (95 % CI = 337.4 to 349.0) as presented in Table 2.

**Table 2:** Changes in intervention effects (difference^a^) for calcium intake results at 1st and 2nd follow-up at cluster level: unadjusted versus adjusted and with versus without exclusions

	Comparison of intervention effect (difference) using summaries of unadjusted means			Comparison of intervention effect (difference) using summaries of unstandardized residuals^c^								
				No exclusion			Exclude 2SD outliers^b^			Exclude 2SD outliers and adjusted for socioeconomic factors^d^		
	Difference^a^	95 % CI	*p*	Difference^a^	95 % CI	*p*	Difference^a^	95 % CI	*p*	Difference^a^	95 % CI	*p*
1st Follow-up	401.15	395.9–406.4	<0.0005	400.2	395.0–405.4	<0.0005	355.5	349.4–361.7	<0.0005	343.2	337.4–349.0	<0.0005
2nd Follow-up	366.7	360.6–372.8	<0.0005	368.8	363.0–374.6	<0.0005	317.0	311.1–322.8	<0.0005	290.5	285.3–295.7	<0.0005

^a^Difference = difference between mean baseline and follow up measures

^b^12 cases at 1st follow-up and 14 cases at second follow-up

^c^Adjusted for baseline values; ^d^Personal income, education and religion

At the second follow up (6 months), the mean difference in calcium intake was 368.8 mg/day (95 % CI = 363.0 to 374.6) without cluster-level adjustment for potential confounders. This fell to 317.0 mg/day (95 % CI = 311.1 to 322.8) after exclusion of data outside two standard deviations. After adjustment for potential confounders, the mean difference was 290.5 mg/day (95 % CI = 285.3 to 295.7) (Table 2).

#### Load -bearing moderate to vigorous physical activity (MVPA)

At the first follow up, the mean difference in load-bearing MVPA was 71.8 min/week (95 % CI = 56.2 to 87.5) without adjustment for potential confounders. This fell to 61.3 min/week (95 % CI = 60.3 to 62.2) after exclusion of data outside two standard deviations. As for the calcium intake outcome, exclusion of two standard deviation outliers in a sensitivity analysis (detailed in Additional file 2 “Sensitivity analysis report”) led to the most conservative estimates of the intervention effect. After adjustment for potential confounders, the mean difference was 55.6 min/week (95 % CI = 54.5 to 56.6) (Table 3).

**Table 3:** Changes in intervention effects (difference^a^) for load-bearing moderate to vigorous physical activity (MVPA) results at 1st and 2nd follow-up at cluster level

	Comparison of intervention effect (difference) using summaries of unadjusted means			Comparison of intervention effect (difference) using summaries of unstandardized residuals^c^								
				No exclusion			Exclude 2SD outliers^b^			Exclude 2SD outliers and adjusted for socioeconomic factors^d^		
	Difference^a^	95 % CI	p	Difference^a^	95 % CI	*p*	Difference^a^	95 % CI	*p*	Difference^a^	95 % CI	*p*
1st Follow-up	77.6	74.9 – 80.3	<0.0005	71.8	56.2–87.5	<0.0005	61.3	60.3–62.2	<0.0005	55.6	54.5–56.6	<0.0005
2nd Follow-up	76.4	73.7–79.1	<0.0005	74.2	71.8–76.5	<0.0005	51.2	49.5–52.9	<0.0005	50.9	49.3–52.6	<0.0005

^a^Difference = difference between mean baseline and follow up measures; ^b^25 cases at 1st follow-up and 21 cases at 2nd follow-up

^c^Adjusted for baseline values; ^d^Personal income, education and religion

At the second follow up, the mean difference was 74.2 min/week (95 % CI = 71.8 to 76.5) without adjustment for potential confounders. This fell to 51.2 min/week (95 % CI = 49.5 to 52.9) after exclusion of data outside two standard deviations. There was a small attenuation to 50.9 min/week (95 % CI = 49.3 to 52.6) after adjustment for potential confounders (Table 3).

This study took a conservative baseline observation carry forward approach to examine the impact of missing data on the intervention effect for both outcome measures. The sensitivity analysis reported attenuation when the missing data were replaced with baseline values but the intervention effect for both calcium intake and load-bearing MVPA remained favourable and statistically significant. This output for this sensitivity analysis is available in Additional file 2 “Sensitivity analysis report”.

#### Dichotomous analysis of calcium intake and load-bearing MVPA data

Dichotomous analysis showed a significantly higher proportion of participants in the intervention group meeting the calcium RDA when compared to the control group. At the first follow-up, there are 47 % more participants in the intervention group who met the calcium RDA compared to the control group and this dropped to 37.8 % at the second follow-up (Table 4).

**Table 4:** Comparing percentage meeting calcium RDA (%): intervention versus control clusters

	Difference	95 % CI	*p*
Baseline	−0.68	−8.6–7.2	0.86
1st follow-up	47.0	39.6–54.4	<0.0005
2nd follow-up	37.8	28.5–47.0	<0.0005

Dichotomous analysis for physical activity also showed a significant difference between the intervention and control group. At baseline, there was minimal difference between both groups (Table 5). At the first follow up, the intervention group reported 30.9 % less participants who reported nil load-bearing MVPA compared to control group and this fell to 10.9 % at second follow-up (Table 5). At the first follow up, the intervention group has 40.3 % more participants than the control group who reported 60 min or more load-bearing MVPA per week and this fell to 33.1 % at the second follow-up (Table 5).

**Table 5:** Comparing percentage reporting nil and ≥ 60 min of leisure time load-bearing moderate to vigorous physical activity (MVPA): intervention versus control clusters

Comparing nil leisure time load-bearing MVPA per week (%)			
	Difference	95 % CI	*p*
Baseline	3.7	−8.1–15.5	0.51
1st follow-up	−30.9	−38.3– − 23.6	<0.0005
2nd follow-up	−10.9	−8.1–15.5	0.04
Comparing ≥ 60 min of leisure time load-bearing MVPA per week (%)			
	Difference	95 % CI	*p*
Baseline	0.1	−9.9–10.1	0.99
1st follow-up	40.3	28.6–52.0	<0.0005
2nd follow-up	33.1	19.7–46.5	<0.0005

#### Intracluster correlation coefficients (ICC)

The post hoc ICCs (ρ) with 95 % confidence intervals for the two major outcomes were daily calcium intake = 0.0951 (0.051, 0.139), and load-bearing MVPA = 0.0040 (−0.007, 0.015).

## Discussion

This cluster randomized trial of the efficacy of a workplace osteoporosis prevention intervention showed significantly greater improvement in calcium intake and load-bearing MVPA in the intervention compared with the control group. Though these improvements attenuated to some degree at the 6-month follow-up, they continued to be significantly and substantially better for intervention versus control groups. The magnitude of change in both calcium intake and physical activity was greater than that reported in previous studies addressing osteoporosis prevention behaviours.

Calcium intake improvements in the intervention group attenuated to 300 mg 6 months post intervention but this is still valuable from a public health perspective as the population attributable fraction of hip fracture has been estimated to decrease by 3.34 % with every 300 mg increase in calcium [4]. This increase is particularly valuable for women who have low calcium intake. In our study cohort, the mean calcium intake at baseline was 458.5 mg. About a quarter of osteoporotic fractures are independently attributable to low dietary calcium intake (<391 mg/day) [40]. In our study, 50 % of the women reported calcium intake under 391 mg/day at baseline. The reported osteoporotic fracture relative risk (RR) is 1.66 for a low dietary calcium intake (<=391 mg/day) when compared to a higher intake (>/=648 mg/day) [40]. Our study saw an almost two-fold increase in the proportion of women who met calcium RDA (800 mg) in the intervention group. The intervention strategies implemented in this study have demonstrated the potential to reduce women’s risk of osteoporosis and osteoporotic fractures appreciably.

The study targeted the specific measure of physical activity - moderate to vigorous load bearing physical activity - that has been proven to affect bone mass and osteoporosis prevention. The increase in load-bearing MVPA in the intervention group was 63.6 min greater than the control group immediately after the intervention and 51.2 min 6 months later. The risk of hip fracture declines 6 % for every increase of three MET hours/week, which is equivalent to 60 min per week walking at an average pace [41]. If sustained, the increase in load bearing MVPA observed in this study has the potential to decrease fracture risk. The decision to target and measure load bearing MVPA makes it challenging to find comparable studies. Existing studies on osteoporosis prevention behaviours are not comparable in experimental design and methodological rigor. Most measure a less specific domain of physical activity. When designing the intervention, this study drew on recommendations from meta-analyses of general physical activity interventions. Despite targeting such a specific domain of physical activity, the intervention group showed results comparable with other well-designed physical activity studies targeting less specific types of activities [42, 43]. The improvement in load-bearing MVPA, compared with the control group, was similar to the improvement in MVPA reported in two other well-designed workplace studies with similar intervention strategies [42, 43]. A possible explanation is that the majority of the MVPA reported in our study falls under the subset of load-bearing MVPA, which means that the load-bearing MVPA in our cohort is representative of the general MVPA.

### Strengths

This study has several strengths. First is the strong study design with a control group who only received print resources and individual calcium feedback without the behavioural strategies. To our knowledge, this is the first osteoporosis prevention behaviour study to use a cluster randomized design conducted and analyzed in accordance with CONSORT guidelines. This study design eliminated the problem of contamination, which is important for an intervention that has a strong social component. The study design incorporated a highly conservative data analysis strategy to account for clustering effect by comparing cluster summaries instead of analyzing individual values. It adopted the approach of excluding values outside two standard deviations and adjusting for baseline outcome values and socioeconomic factors. The results stood up to the rigor of this approach and the intervention effects remained statistically and clinically significant. Attenuation for both outcome measures was higher with exclusion of outliers than with adjustment for socioeconomic factors. This is likely due to over-reporting in a small number of subjects in the intervention group.

Another strength of this study is the integration of evidence-based elements into the intervention design to develop a strong behavioural strategies, rather than cognitive strategies, for osteoporosis prevention [14, 25, 26, 32, 33]. Diet and physical activity were treated as unique behaviours and were addressed using unique evidence-based intervention strategies.

A novel component of the study was the utilization of qualitative dietary information at baseline to guide the intervention. It was noted that women who already included a wide range of calcium-rich foods in their diet were still unable to meet the RDA due to a shortfall in quantity. The amount of calcium in a serving of food can be a very abstract concept, which is challenging to convey through mainstream media campaigns. Participants in the intervention group were supported to achieve sufficient quantity without substantial changes to their dietary habits. Local food sampling was a very economical and efficacious exercise to address portion sizes and common barriers such as taste aversion and cost concerns. It is worth noting that the control group subjects also received feedback on their calcium intake accompanied with education resources but made minimal improvements in their intake.

This study incorporated cumulative 5–10 min blocks of load-bearing MVPA, in between or during daily routines to address the barrier of time. Daily short duration load-bearing activities of adequate intensity have been proven to increase bone mineral density especially in pre-menopausal women [44, 45]. Evidence points to other long-term physiological benefits from short duration load-bearing MVPA for sedentary adults [46, 47] who are more likely to adhere to this pattern of exercise [46, 47].

The focus on ownership of individual’s goals and behaviour strategies, and the encouragement of incorporating changes with minimal disruption to existing lifestyle may have limited the attenuation in physical activity outcome after 6 months.

To our knowledge, this first osteoporosis prevention behaviour change study utilized the worksite as a delivery platform. While individual goal setting and problem solving was the central component of the intervention, the delivery platform was chosen for its capacity to incorporate social models to facilitate behaviour change. This is a possible explanation for stronger outcomes compared to previous community-based cluster physical activity trials that intervened primarily at the individual level.

### Limitations

While a strong randomized controlled design was used for this study, there are elements that could have been better controlled for a more stringent comparison, such as providing the same level of attention to both groups with variation only in the content [48]. The presence of a control with “attention intervention” is recommended for future studies to examine the efficacy of the intervention described more rigorously.

Another limitation of this study was the non-availability of bone mineral density as an outcome measure. The outcomes measures in this study were derived from self-reports, which may incur potential bias. Steps were taken in this study to ensure the self-report measurement tools used were valid and reliable for the study population. Nevertheless, the osteoporosis preventive potential of the intervention would be more accurately and precisely assessed using objective bone mass density or other biomarkers. The observed changes in physical activity behaviour in this study may have led to improvements in site-specific bone mineral density such as in the hips [44, 49]. Objective outcome measures would also have eliminated the precision and accuracy issues posed by self-reported outcome measures. However, resource and feasibility limitations excluded these possibilities. The positive results of the present study might help to justify the use of bone mineral density measurements in future trials with longer duration of follow up.

Not targeting workplace policies and working conditions as part of the intervention was a third limitation. Inclusion of organization-level actions would have complemented and better supported individual behaviour change [50] but it would have been harder to recruit worksites into the study and would have entailed greater complexity in the intervention process. Future studies could integrate job and work station modifications such that both work-related and non-work-related risk and protective factors, with the potential to further improve the prevention of osteoporosis as well as other chronic diseases [50, 51]. Collection of individual process data in future studies is also recommended so the efficacy of different components within the intervention can be examined.

Future studies could also extend follow-up beyond 6 months to assess the effect sustainability. At the time of the study, published ICC values for the targeted outcome and mediators were either limited or absent. In retrospect, it is now known that ICCs were overestimated in the sample size calculations. The ICC estimates published in this study can be used in the design of future intervention studies in this area.

Potential selection bias due to subject attrition is a common limitation in population-based studies. This study reported loss of subjects due to work factors such as resignation and corporate restructuring, and due to personal factors like pregnancy. Over-recruitment of subjects within cluster subjects was a strategy used to maintain cluster sample size.

Another limitation is that this study did not have sufficient data about the non-responders to compare with the participants in this study. This means that the conclusions cannot be generalised to the general female working population from which our sample was taken. The objective of this study was to investigate the efficacy of a targeted intervention that focused on behavioural strategies. Future research should progress to effectiveness trials that incorporate methodological strategies for reporting on external validity data.

## Conclusions

The women in the intervention group reported significant and meaningful improvements in both calcium intake and physical activity that may have a positive and measurable impact on the risk of osteoporotic fracture. Considering the ease of widespread dissemination at workplaces, additional studies should progress to investigate the effectiveness of this intervention on women from a more diverse sample of workplaces, including bone mineral density or other objective outcomes to complement behavioural outcome measures.

## Additional files

Additional file 1: Physical activity definition. (PDF 169 kb) Additional file 2: Result description and Sensitivity analysis report. (PDF 221 kb)
