# Supplementary material for: Efficacy of a workplace osteoporosis prevention intervention: a cluster randomized trial
Source: BMC Public Health. 2016 Aug 24;16(1):859. doi: 10.1186/s12889-016-3506-y (PMC4995796; doi:10.1186/s12889-016-3506-y)
Supplement: Additional file 1: — Physical activity definition. (PDF 169 kb) [file 12889_2016_3506_MOESM1_ESM.pdf]

Supplementary material:

Definition of load-bearing moderate to vigorous intensity physical activity (Load-bearing MVPA)

Title of manuscript:

Efficacy of a workplace osteoporosis prevention intervention: a cluster randomised trial.

Authors:

Ai May Tan
Anthony D. LaMontagne
Dallas R English
Peter Howard

Corresponding author:

Ai May Tan *, BSc (Phty), MAppSc (Phty)
McCaughey Centre: VicHealth Centre for the Promotion of Mental Health & Community Wellbeing
Melbourne School of Population and Global Health
University of Melbourne, VIC 3010 AUSTRALIA
Email: amtan@student.unimelb.edu.au

Definition of load-bearing moderate to vigorous intensity physical activity (Load-bearing MVPA)

Load-bearing physical activity (also known as weight-bearing physical activity) refers to a physical activity during which the body works against the force of gravity and the feet and legs carry a person's weight. Examples include walking, jogging and dancing.

Moderate and vigorous physical activity

A metabolic equivalent, or MET, is a unit useful for describing the energy expenditure of a specific activity. A MET is the ratio of the rate of energy expended during an activity to the rate of energy expended at rest. For example, 1 MET is the rate of energy expenditure while at rest. A 4 MET activity expends 4 times the energy used by the body at rest. If a person does a 4 MET activity for 30 minutes, he or she has done 4 × 30 = 120 MET-minutes (or 2.0 MET-hours) of physical activity. A person could also achieve 120 MET-minutes by doing an 8 MET activity for 15 minutes [1, 2].

The US Department of Health and Human services defined absolute aerobic intensity in terms of METs and this definition is also adopted by WHO Global Recommendation on Physical Activity 2010 [2].

Moderate-intensity activities are defined as 3.0 to 5.9 METs. Walking at 3.0 miles per hour requires 3.3 METs of energy expenditure and is therefore considered a moderate-intensity activity. On an absolute scale, moderate intensity refers to the physical activity that is performed at 3.0–5.9 times the intensity of rest. On a scale relative to an individual's personal capacity, moderate-intensity physical activity is usually a 5 or 6 on a scale of 0–10 [1, 2].

Vigorous-intensity activities are defined as 6.0 METs or more. Running at 10 minutes per mile (6.0 mph) is a 10 MET activity and is therefore classified as vigorous intensity. On an absolute scale, vigorous intensity refers to physical activity that is performed at 6.0 or more times the intensity of rest for adults and typically 7.0 or more times for children and youth. On a scale relative to an individual's personal capacity, vigorous-intensity physical activity is usually a 7 or 8 on a scale of 0–10 [1, 2].

Reference:

1. **Global Recommendations on Physical Activity for Health**. World Health Organization, Geneva, 2010.
2. **2008 Physical Activity Guidelines for Americans**. Office of Disease Prevention & Health Promotion UDoHaHS, October 2008.
