# Supplementary material for: Efficacy of a workplace osteoporosis prevention intervention: a cluster randomized trial
Source: BMC Public Health. 2016 Aug 24;16(1):859. doi: 10.1186/s12889-016-3506-y (PMC4995796; doi:10.1186/s12889-016-3506-y)
Supplement: Additional file 2: — Result description and Sensitivity analysis report. (PDF 221 kb) [file 12889_2016_3506_MOESM2_ESM.pdf]

Supplementary material:

1. Description of observed outcome measures at baseline, 1st follow-up and 2nd follow-up (box plot)

2. Sensitivity analysis report

Title of manuscript:

Efficacy of a workplace osteoporosis prevention intervention: a cluster randomised trial.

Authors:

Ai May Tan
Anthony D. LaMontagne
Dallas R English
Peter Howard

Corresponding author:

Ai May Tan *, BSc (Phty), MAppSc (Phty)
McCaughey Centre: VicHealth Centre for the Promotion of Mental Health & Community Wellbeing
Melbourne School of Population and Global Health
University of Melbourne, VIC 3010 AUSTRALIA
Email: amtan@student.unimelb.edu.au

1. **Description of observed outcome measures at baseline, 1st follow-up and 2nd follow-up (box plot)**

Box plots comparing the unadjusted mean calcium intake values for both intervention and control group at baseline and at first and second follow up.

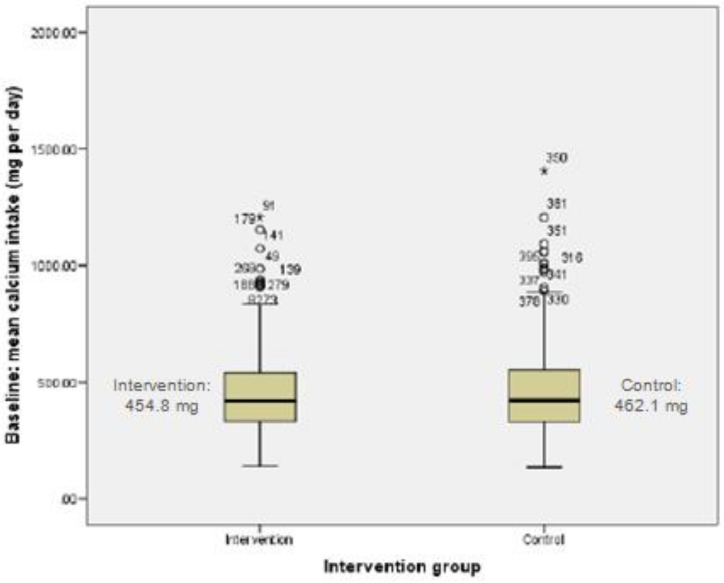

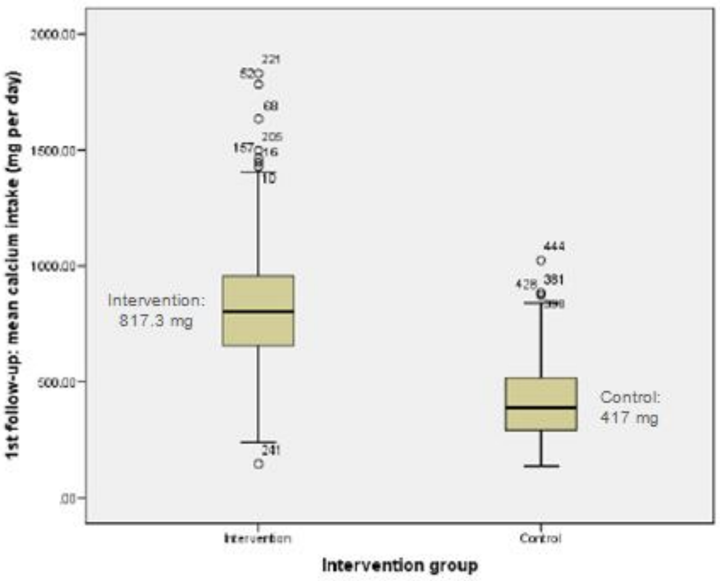

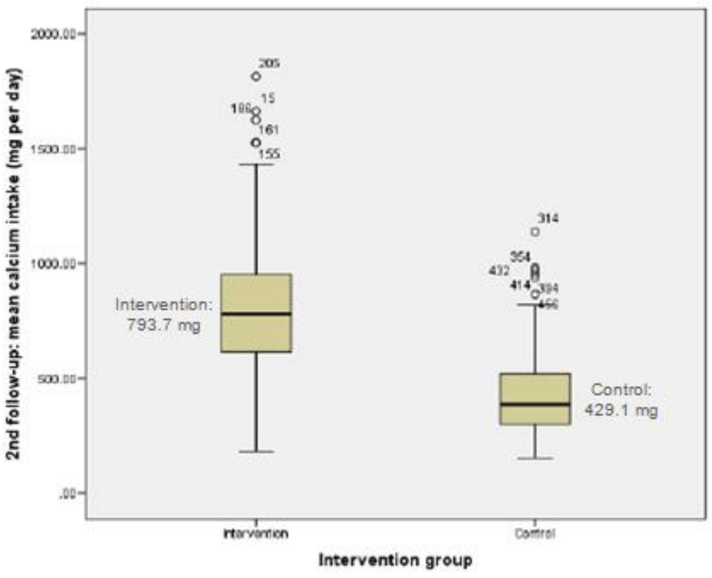

Box plots comparing the unadjusted mean load-bearing moderate to vigorous physical activity for both intervention and control group at baseline and at first and second follow up.

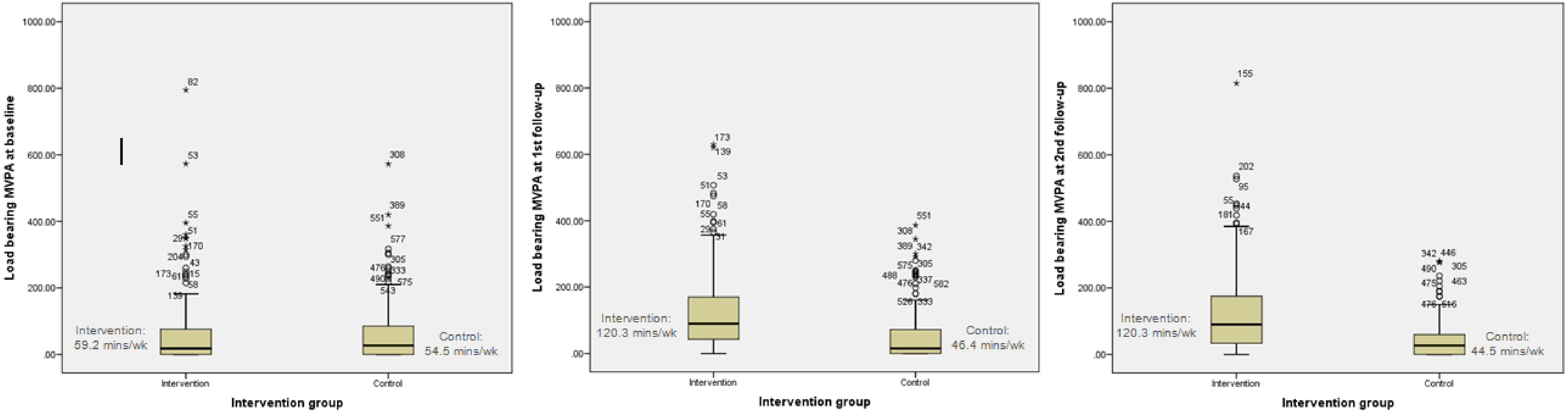

2. Sensitivity analysis report

a. Effects of extreme values

Sensitivity analysis was conducted to examine the effects of extreme values on the intervention effect for calcium intake and load-bearing moderate to vigorous physical activity (MVPA). The analysis examined the effects of excluding two and three standard deviation (SD) outliers and the effects of raw data truncation at 95th percentile and 99th percentile, adjusting only for baseline (Table 1).

At the first follow-up, the intervention effect for load-bearing MVPA without any exclusion or truncation was 71.8 minutes/week, 95% CI [56.2, 87.5]. All exclusions and truncation led to attenuation.

The greatest attenuation was observed with exclusion of two standard deviation outliers where the intervention effect fell to 61.3 minutes/week, 95% CI [60.3, 62.2].

Exclusion of three SD outliers and truncation at 95th percentile resulted in decrease in intervention effect to 68.1 and 66.5 minutes/week respectively. Truncation at 99th percentile resulted in the minimal attenuation at 71.6 minutes/week.

The outcome of the analysis for calcium intake is similar. The intervention effect without exclusion and truncation is 400.2 mg/day, 95% CI [395, 405]. The attenuations were small and the largest decrease was reported with the exclusion of two standard deviation outliers at 355.5 mg/day, 95% CI [349, 362]. The intervention effects for exclusion of three standard deviation outliers and truncation at 95th and 99th percentile were 377, 376 and 396 mg/day respectively.

Sensitivity analysis at the second follow-up also reported largest degree of attenuation with exclusion of two standard deviation outliers. At the second follow-up, the intervention effect for load-bearing MVPA without any exclusion or truncation was 74.2 minutes/week, 95% CI [71.8, 76.5]. All exclusions and truncation led to attenuation. The greatest attenuation was observed with exclusion of two SD outliers where the intervention effect fell to 51.2 minutes/week, 95% CI [49.5, 52.9]. Exclusion of three SD outliers and truncation at 95th percentile resulted in similar

changes in intervention effect at 66.1 and 66.2 minutes/week respectively. Truncation at 99th percentile resulted in the least attenuation at 72.5 minutes/week.

The intervention effect for calcium intake without any exclusion or truncation is 369 mg/day, 95% CI [363, 375]. Exclusion of two standard deviation outliers led to the largest attenuation to 317 mg/day, 95% CI [311, 323]. The intervention effects for exclusion of three SD outliers and truncation at 95th and 99th percentile are 345, 347 and 367 mg/day respectively.

The sensitivity analysis consistently demonstrated that exclusion of two standard deviation outliers led to the highest degree in attenuation in the intervention effect for both outcomes measures. The most conservative approach by excluding measurements that fell outside two standard deviations is recommended.

This excluded 12 and 14 subjects from calcium intake analysis, all from the intervention group, at first and second follow up respectively. This also excluded 12 and 14 subjects from the physical activity analysis data. In the first follow-up, 22 were from the intervention group and three from control group. In the second follow-up 18 were from the intervention group and three from control.

b. Effects of baseline observation carry forward to replace missing observations

Sensitivity analysis was conducted to examine the effects of missing values on the intervention effect for calcium intake and load-bearing moderate to vigorous physical activity (MVPA). This analysis adopted the baseline observation carry forward approach, which means that all missing data in the first and second follow up were replaced by the baseline data. The same two stage analysis, as described in the result section, was carried out to assess the impact on the intervention.

The intervention effect for calcium with exclusion of two standard deviation outliers and baseline adjustment was 355.5 mg/day, 95% CI [349.4, 361.7] at the first follow-up and 317.0 mg/day, 95% CI [311.1, 322.8] at second follow-up, both being highly significant. The replacement of missing data with baseline data saw an attenuation to

255.6 mg/day, 95% CI [220.3, 230.9] at the first follow up and 198.4mg/day, 95% CI [193.2, 203.6] at second follow up. The results are displayed in Table 3. There was a moderate to large attenuation but the intervention effect stayed highly significant.

The intervention effect for load-bearing MVPA with exclusion of two standard deviation outliers and baseline adjustment was 61.3 minutes/week, 95% CI [60.3, 62.2] at the first follow-up and 51.2 minutes/week, 95% CI [49.5, 52.9] at second follow-up, both being highly significant. The replacement of missing data with baseline data saw an attenuation to 51.8 minutes/week, 95% CI [50.9, 52.7] at the first follow up and 40.7 minutes/week, 95% CI [39.7, 41.7] at second follow up. The results are displayed in Table 3. There was a moderate attenuation but the intervention effect stayed highly significant.

Table 1 Sensitivity analysis to assess the influence of data exclusion and truncation at 1st follow-up

		Mean Difference	95% Confidence Interval of the Difference		*p*
			Lower	*Upper*	
Comparing cluster means of load-bearing MVPA residuals (covariate: baseline)	No exclusion or truncation	71.8	56.2	87.5	<0.0005
	Exclude 2SD outliers	61.3	60.3	62.2	<0.0005
	Exclude 3SD outliers	68.1	56.8	79.5	<0.0005
	Data truncated at 99th percentile	71.6	58.2	84.9	<0.0005
	Data truncated at 95th percentile	66.5	55.2	77.9	<0.0005
Comparing cluster means of calcium intake residuals (covariate: baseline)	No exclusion or truncation	400.2	395.0	405.4	<0.0005
	Exclude 2SD outliers	355.5	349.4	361.7	<0.0005
	Exclude 3SD outliers	376.8	370.9	382.7	<0.0005
	Data truncated at 99th percentile	396.2	390.9	401.4	<0.0005
	Data truncated at 95th percentile	375.5	370.4	380.7	<0.0005

Table 2 Sensitivity analysis to assess the influence of data exclusion and truncation at 2nd follow-up

		Mean Difference	95% Confidence Interval of the Difference		*p*
			Lower	*Upper*	
Comparing cluster means of load-bearing MVPA residuals (covariate: baseline)	No exclusion or truncation	74.2	71.8	76.5	<0.0005
	Exclude 2SD outliers	51.2	49.5	52.9	<0.0005
	Exclude 3SD outliers	66.1	64.1	68.1	<0.0005
	Data truncated at 99th percentile	72.5	70.4	74.6	<0.0005
	Data truncated at 95th percentile	66.2	64.3	68.0	<0.0005
Comparing cluster means of calcium intake residuals (covariate: baseline)	No exclusion or truncation	368.9	363.0	374.6	<0.0005
	Exclude 2SD outliers	317	311.1	322.8	<0.0005
	Exclude 3SD outliers	345	339.6	350.4	<0.0005
	Data truncated at 99th percentile	366.6	360.6	372.5	<0.0005
	Data truncated at 95th percentile	347.4	342.1	352.8	<0.0005

Table 3 Sensitivity analysis to assess the impact of replacing missing data with baseline observations at 1st and 2nd follow up

			Mean Difference	95% Confidence Interval of the Difference		*p*
				Lower	*Upper*	
Comparing cluster means of calcium intake residuals (covariate: baseline) exclude 2 SD outlier	Missing data not replaced	1st follow-up	355.5	349.4	361.7	<0.0005
		2nd follow-up	317.0	311.1	322.8	<0.0005
	Missing data replaced by baseline observation	1st follow-up	255.6	220.3	230.9	<0.0005
		2nd follow-up	198.4	193.2	203.6	<0.0005
Comparing cluster means of load-bearing MVPA residuals (covariate: baseline) exclude 2SD outlier	Missing data not replaced	1st follow-up	61.3	60.3	62.2	<0.0005
		2nd follow-up	51.2	49.5	52.9	<0.0005
	Missing data replaced by baseline observation	1st follow-up	51.8	50.9	52.7	<0.0005
		2nd follow-up	40.7	39.7	41.7	<0.0005
